# Supplementary material for: Polyketide synthase gene clusters in the ericoid mycorrhizal fungus Oidiodendron maius: genome-wide prediction, expression profiling, and functional characterization of knockout mutants
Source: Front Plant Sci. 2026 Sep 16;17:1924430. doi: 10.3389/fpls.2026.1924430 (PMC13623903; doi:10.3389/fpls.2026.1924430)
Supplement: Supplementary file 1 [file SupplementaryFile1.zip › Supplementary Materials/Supplementary Folder 1.zip!/Oidma1_AssemblyScaffolds_Repeatmasked/knownclusterblast/cluster51/jgi.p_Oidma1_137730_mibig_hits.html]

| MIBiG Protein | Description | MIBiG Cluster | MiBiG Product | % ID | % Coverage | BLAST Score | E-value |
| --- | --- | --- | --- | --- | --- | --- | --- |
| CBL93725.1 | 3-oxoacid\_CoA-transferase | BGC0000360 | GE81112\_biosynthetic\_gene\_cluster | 57 | 44.2687747036 | 236 | 6.1e-62 |
| ABS74184.1 | ScoB | BGC0001090 | Bacillomycin\_biosynthetic\_gene\_cluster | 56 | 42.6877470356 | 234 | 3e-61 |
| BAB69695.1 |  | BGC0001098 | Iturin\_biosynthetic\_gene\_cluster | 56 | 42.6877470356 | 234 | 3e-61 |
| AAK53493.1 | beta-subunit\_of\_putative\_3-oxoacid\_CoA-transferase | BGC0000774 | Lipopolysaccharide\_biosynthetic\_gene\_cluster | 56 | 41.3043478261 | 223 | 7e-58 |
| BAB69694.1 |  | BGC0001098 | Iturin\_biosynthetic\_gene\_cluster | 46 | 49.8023715415 | 210 | 6.1e-54 |
| ABS74185.1 | ScoA | BGC0001090 | Bacillomycin\_biosynthetic\_gene\_cluster | 46 | 49.8023715415 | 208 | 1.8e-53 |
| AAK53462.1 | alpha\_subunit\_of\_putative\_3-oxoacid\_CoA-transferase | BGC0000774 | Lipopolysaccharide\_biosynthetic\_gene\_cluster | 38 | 50.9881422925 | 175 | 2.2e-43 |
| AAV37065.1 | CoA\_transferase\_subunit\_A | BGC0000015\_c2 | Amphotericin\_biosynthetic\_gene\_cluster | 39 | 45.652173913 | 155 | 2.3e-37 |
| AAV37066.1 | CoA\_transferase\_subunit\_B | BGC0000015\_c2 | Amphotericin\_biosynthetic\_gene\_cluster | 41 | 41.3043478261 | 146 | 6.4e-35 |
